# Delivering Evidence-Based Online Concussion Education to Medical and Healthcare Professionals: The Concussion Awareness Training Tool (CATT)

**DOI:** 10.1155/2020/8896601

**Published:** 2020-12-22

**Authors:** Shelina Babul, Kate Turcotte, Maude Lambert, Gabrielle Hadly, Karen Sadler

**Affiliations:** ^1^BC Injury Research and Prevention Unit, BC Children's Hospital, Vancouver, Canada; ^2^Department of Pediatrics, Faculty of Medicine, University of British Columbia, Vancouver, Canada; ^3^School of Psychology, Behavioural Neurosciences, University of Ottawa, Ottawa, Canada; ^4^School of Population & Public Health, University of British Columbia, Vancouver, Canada

## Abstract

**Background:**

Medical and healthcare professionals report an important gap in their training and knowledge on concussion diagnosis and management. The Concussion Awareness Training Tool (CATT) for medical professionals provides evidenced-based training and resources, representing an important effort to fill this gap. The goal of the current article was to summarize and describe the general uptake of the 2018 relaunch of the CATT for medical professionals and to present results of a quality assurance/quality improvement (QA/QI) assessment including qualitative feedback from medical and healthcare professionals. *Methodology*. Tracking completions via certificates and Google Analytics were used to measure uptake over the first two years following the 2018 relaunch and promotion of CATT for medical professionals. Medical and healthcare professionals who had completed the CATT from the time of the relaunch on June 11, 2018, to July 31, 2019, were invited via e-mail to participate in the survey-based QA/QI assessment. Both quantitative and qualitative data were collected.

**Results:**

Year 1 saw 8,072 pageviews for the CATT for medical professionals landing page, increasing to 9,382 in Year 2. Eighty-nine medical and healthcare professionals who had completed the CATT for medical professionals participated in the QA/QI assessment. Results showed that 85% of respondents reported learning new information about concussion; 73% reported changing the way they diagnose, treat, or manage concussion; and 71% reported recommending the CATT to colleagues. Qualitative data also indicated highly favourable opinions and experiences.

**Conclusions:**

The CATT for medical professionals has demonstrated promise as a tool to promote knowledge translation practice and help fill the gap in concussion training and knowledge reported by medical and healthcare professionals.

## 1. Introduction

Concussion, a mild traumatic brain injury (TBI), is the most common type of neurologic disorder, accounting for 70–90% of all traumatic brain injuries [1]. Circumstances leading to a concussion incident are diverse as it can happen to anyone, at any time during sport, work, play, or regular daily activities. In sport medicine, concussion is considered to be one of the most complex injuries to assess, diagnose, and manage, mainly because of the rapidly evolving onset and diverse symptoms [2].

The global economic burden related to concussion and TBI is estimated to approximately $400 billion annually, representing a considerable portion of the global injury burden worldwide [3]. Annual lifetime cost of TBI is estimated to $945 million, 31% going into medical treatment costs and 69% into lost productivity costs [4]. This heavy financial burden on the healthcare system may be exacerbated by a lack of concussion training, limiting medical and healthcare professionals' abilities to employ best practices for diagnosis and management, thereby leading to unnecessary referrals to specialists and longer recovery time [5, 6]. Additionally, important deficiencies in the concussion curriculum of Canadian and American medical schools have been identified in several studies [6–9]. Although the majority of medical students and residents are able to correctly define what a concussion is, they have difficulties correctly identifying concussion symptoms and optimal diagnosis and management strategies, lack the practical experience with concussion diagnosis and treatment, and have never attended a lecture about concussion during their university training [6]. Similarly, a significant proportion of physicians provide concussion recommendations that are not in line with current evidence-based guidelines [10, 11]. For example, recent changes in concussion treatment best practices include resting with limited cognitive and physical activity for 24 to 48 hours after injury, yet several physicians report not consistently recommending any cognitive or physical rest after a concussion [10, 12]. Studies also suggest that physicians employ neuroimaging in concussion diagnosis significantly more frequently than recommended by evidence-based guidelines. These unnecessary scans not only significantly increase the concussion-related financial burden but also expose patients to excessive radiation [13, 14]. This substantial knowledge gap among medical and healthcare professionals is also worrisome given that failure to properly treat and manage concussion can lead to complications and delayed recovery [6, 11]. For instance, failure to follow best practices can significantly increase the risk of second-impact syndrome (SIS), a rare but disastrous traumatic brain injury occurring when a second head injury is sustained before the first concussion has resolved [15]. The mortality rate for SIS is almost 50%, and the disability rate among those who survive is close to 100% [16, 17]. Encouragingly, an overwhelming majority of residents and medical professionals indicate a desire and interest to pursue concussion management training with up-to-date evidence-based information [6, 10].

Given that emergency and general practice family physicians and nurses are often the first line of medical and healthcare professionals providing assessment for patients with suspected concussion [18], specialized training on the latest evidence-based practices in concussion diagnosis and management is crucial in this population [5]. Harmonizing concussion education and providing evidence-based up-to-date training for concussion diagnosis and management would offer adequate support to medical and healthcare professionals, improve abilities to provide optimal patient care, and represent a potential step forward towards the reduction of the concussion-related financial burden in our society [8, 13, 19].

The online Concussion Awareness Training Tool (CATT; https://cattonline.com/) for medical professionals, originally launched in 2013, was developed to fulfil this gap in the education training of medical and healthcare professionals. Its content is based upon international research, expert consultation, and extensive external review and feedback [20]. The original CATT for medical professionals consisted of a 40-minute e-learning module with a wide range of supplementary resources for concussion assessment and management, such as online concussion assessment tools, standardized clinical assessment guidelines, return to activity strategies, printable resources for patients, peer-reviewed journal articles, and specific videos on how to perform cranial nerve exams. The 2013 evaluation survey (pre-/postintervention questionnaire design) revealed promising results. Indeed, after completing the CATT module, both physicians (*p* < 0.01) and nurses (*p* < 0.05) were found to have a statistically significant positive change in their practices, such as using evidence-based guidelines in making concussion treatment decisions, recommending both physical and cognitive rest, and providing patients with concussion information or resources [21, 22]. Physicians who reported treating more than 10 concussions per year also demonstrated a significant positive change in their concussion-related knowledge (*p* = 0.04) [21, 22], which was assessed by asking a series of questions related to the Consensus Statement on Concussion in Sport, concussion symptoms and timeline, requirements for concussion diagnosis, and recommended concussion treatment and management strategies.

When initially launched in 2013, the overarching aim of the CATT for medical professionals was to serve as a general awareness tool, ensuring concussions were taken seriously, immediately recognized, and accurately diagnosed by all healthcare professionals. Minimal information was provided with regard to concussion treatment and management. Over the years, as the general awareness increased around this “invisible” injury and science and guidelines evolved, the National Concussion Harmonization Project, funded by the Public Health Agency of Canada, identified the need to provide in-depth evidence-based education that could reach a wide audience. The CATT was identified as a recognized and trusted concussion education resource, and the ensuing partnership resulted in a new revised CATT for medical professionals. The 2018 redevelopment content focuses on early clinical assessment and management of concussion during the first two to four weeks postinjury in order to maximizing the patient's opportunity to fully recover. The primary target audience of CATT for medical professionals is emergency and general practice family physicians, but the content is apt for all healthcare professionals involved in the assessment and care of concussion patients, including nurses, paramedics, physiotherapists and athletic therapists, and occupational therapists. The Program Planning Committee was comprised of clinical and nonclinical experts in concussion care and knowledge translation (see Appendix A), and content was developed through an iterative review process. This work was informed by a review of the literature and based primarily upon the Consensus Statement on Concussion in Sport [23], clinical practice guidelines and standards published by the Ontario Neurotrauma Foundation, and the Canadian Guideline on Concussion in Sport [24]. The full list of references is available in Appendix B. The module content was reviewed by Parachute's Concussion Expert Advisory Committee, a past president of Doctors of British Columbia (BC), and selected members of the BC Concussion Advisory Network working in clinical care. The learning objectives of the redeveloped CATT for medical professionals are to

- effectively assess a patient's concussion situation within the initial hours postinjury
- optimally manage concussion care during the first 2–4 weeks postinjury in order to decrease long-term effects; this includes the management of symptoms, and best practices for the return-to-school, work, sport or recreational activities, etc.
- identify when referral to specialty care is required

Organized in five parts, the course content now covers the following:

1. Concussion: definition and epidemiology
2. Medical assessment for concussion, including clinical history, signs and symptoms, physical examination and adjunctive tests, and medical documentation
3. Concussion management and medical clearance, including the role of rest, symptom management, what to inform the patient, returning to activity, and clinical follow-up
4. Persistent concussion symptoms and their management, including making referral decisions
5. The role of physiotherapy and occupational therapy in concussion management

**Figure 1:**
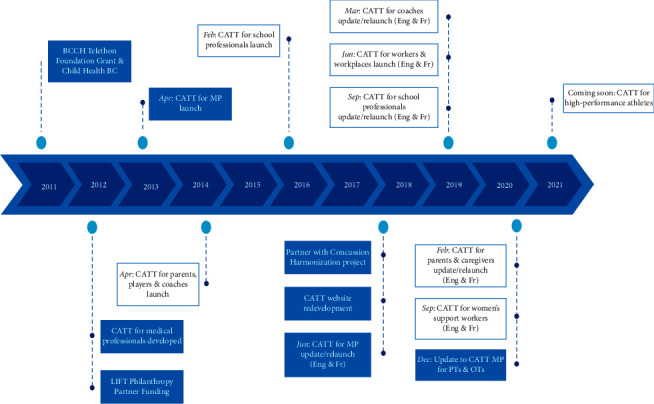
Schematic representation of the timeline of important milestones in the CATT development.

The full content of the module takes approximately two hours to complete. Further details on the content of the CATT for medical professionals module are presented in Table 1. The CATT for medical professionals module is accredited by the University of British Columbia Division of Continuing Professional Development, Faculty of Medicine, and is an Accredited Self-Assessment Program as defined by the Maintenance of Certification Program (MOC) of the Royal College of Physicians and Surgeons of Canada.  Figure 1 provides a schematic representation of the timeline of the CATT activities and allows to specifically situate the CATT for medical professionals module in time amongst the other CATT modules for coaches, parents and caregivers, school professionals, workers and workplaces, women's support workers supporting survivors of intimate partner violence, and high-performance athletes.

The primary aim of this paper is to describe the general uptake of the CATT for medical professionals over its first two years after its relaunch and present the results of a quality assurance/quality improvement (QA/QI) assessment. The QA/QI results serve as an indicator of the quality of the resource and the general receptiveness of medical and healthcare professionals to use it. The current manuscript also includes general information about the CATT for medical professionals (e.g., content and development process).

## 2. Methods

### 2.1. Relaunch and Tracking of the CATT for Medical Professionals

Relaunch of the CATT for medical professionals included the development of a promotional package available in English and French. Primary knowledge translation activities were led by Parachute, the BC Injury Research and Prevention Unit, and BC Children's Hospital.

Completions of the CATT for medical professionals were documented based upon the information required to receive a certificate of completion, that is, first and last name, e-mail address, profession, and geographic information (country, province, and city). Available Google Analytics data for the first two years include the number of visits to the CATT landing page for medical professionals and location from where the resource was accessed. The number of visits to the Sport Concussion Assessment Tool (5^th^ edition; SCAT5), adult and child version, was also monitored. The SCAT5 and Child SCAT5 are widely used, standardized tools for evaluating for a suspected concussion. Developed by the Concussion Group in Sport for use by medical and healthcare professionals, these tools are available with permission on the CATT website as fillable forms to complete and download, compatible with mobile devices.

### 2.2. Study Protocol

All individuals who completed the CATT for medical professionals online education from the time of the launch on June 11, 2018, to July 31, 2019, were invited to participate in the QA/QI assessment. Participants were recruited via the e-mail address they provided in order to receive their CATT completion certificate. Consent was sought and received at the beginning of the QA/QI assessment survey. The survey took approximately 5–10 minutes to complete. Three reminder e-mails for survey completion were sent on September 24, October 8, and October 22, 2019, after which the QA/QI assessment survey was closed on October 31, 2019. The total survey accessibility period lasted just over seven weeks. Approval to conduct this study was provided by the Research Privacy Advisor of the Provincial Health Services Authority of British Columbia. All results of the survey were deidentified.

### 2.3. QA/QI Assessment

The QA/QI assessment survey was provided through REDCap, a secure web application for building and managing online surveys and databases. Participants were invited to complete this 5- to 10-minute survey voluntarily, with all results deidentified.

The survey included a series of three demographic questions and six main questions related to the module: Did you learn new information about concussion diagnosis, treatment, or management from the CATT MP e-learning module? Have you changed the way you diagnose, treat, or manage a patient with concussion since completing the CATT MP e-learning module? Do you access the SCAT5 or the Child SCAT5 from the CATT website to help you assess a patient for concussion? Do you refer your patients to the CATT resource or distribute any CATT materials to them? Have you recommended the CATT e-learning module to other medical professionals? Do you have any further comments about the CATT MP e-learning module?

### 2.4. Data Analysis

Following the end of the survey accessibility period, a descriptive analysis of the quantitative responses was performed and qualitative data were transcribed and summarized by the research team.

## 3. Results

### 3.1. Relaunch and Tracking

The promotional package was developed and disseminated by Parachute, available in English and French, and included a background description, teaser content, full text content, and additional content such as infographics. Sixteen organizations within Canada committed to helping promote the new CATT for medical professionals module (see Appendix C). Promotional activities included social media promotion, newsletter articles, website announcements, and presentations to select audiences.

The CATT for medical professionals was completed by 575 people between the relaunch date of June 11, 2018, and May 31, 2019, and by 628 people in the second year, from June 1, 2019, to May 31, 2020, for a total of 1203 completions. Most people reported residing in Canada (92%). Other countries of residence included the United States, the United Kingdom/Great Britain, Uganda, New Zealand, Australia, Azerbaijan, Brazil, Croatia, Ireland, Israel, Lebanon, Malaysia, Mexico, the Netherlands, Norway, Rwanda, Singapore, South Africa, and the United Arab Emirates.

Profession was provided by 88% of people who completed the CATT for medical professionals module. Among those professions identified, 25% were doctors such as family physicians, sports medicine physicians, pediatricians, and medical students; 16% were first responders such as paramedics, advanced care paramedics, primary care paramedics, emergency first responders, and firefighters; and 16% were nurses including registered nurses, registered nurse practitioners, licenced practical nurses, public health nurses, and students. Other professions included physiotherapists (8%), athletic therapists (7%), occupation therapists (4%), chiropractors (3%), and unidentified students (8%).

Google Analytics for Year 1 indicated 8,072 page views for the CATT for medical professionals landing page, increasing to 9,382 in Year 2. The SCAT5 tool received the most visits, at 18,611 for Year 1 and 24,704 for Year 2 (see Table 2).

### 3.2. QA/QI Study

In total, 654 invitations were sent out through e-mail to the individuals who completed the CATT for medical professionals module from the time of the launch on June 11, 2018, to July 31, 2019. Eight of them were reported as undeliverable, for a total of 646 viable invitations. We received a total of 114 responses; four of these respondents did not consent to participate. Among those who consented to participate, 89 respondents completed the survey in its entirety, resulting in an overall response rate of 13.8%. All respondents reported residing in Canada (primarily from British Columbia, Ontario, or Alberta), and over half had completed the CATT within the last 6 months (52.8%). One-third of respondents indicated that they found out about the CATT from a colleague (32.6%); other common sources included Internet searches (22.5%) and e-mail (19.1%). Respondents' demographic characteristics are detailed in Table 3.

Results from the QA/QI assessment indicated that 85% of respondents reported learning new information about concussion diagnosis, treatment, and management from the CATT for medical professionals module; 73% reported changing the way they diagnose, treat, or manage a patient with concussion following the completion of the CATT for medical professionals module; and 71% reported recommending the CATT to colleagues. More specifically, some respondents reported that the module allowed them to gain a better understanding of what can cause a concussion, the symptoms of concussion, and best practices for optimal treatment (e.g., maximum of two days of rest vs. full rest until full recovery). One respondent stated: “I perform a more detailed assessment than I did previously and I am more likely to refer to a specialist clinic now that I know more about concussion complications.” Many respondents reported that the CATT module provided a “better structure” for concussion management, enhanced effective communication with patients, and made them more aware of available resources. For instance, one respondent said that “getting up-to-date information on intervention techniques, as well as developing resource materials that I could utilize in clinic and give to clients, was very beneficial. It enhanced communication with clients dealing with concussions.”

Additionally, amongst respondents who recently evaluated patients for concussion, 45% reported accessing the SCAT5 or Child SCAT5 from the CATT website, and 48% reported referring patients to CATT online or providing CATT materials (e.g., CATT cards, videos, Return-to-Learn resource). Participants' specific responses to each of the QA/QI survey question are shown in Table 4.

Twenty-five respondents provided additional qualitative feedback. Overall, 21 comments were positive, three were negative, and one was neutral (inquiring about French resources). Overall, respondents expressed highly positive feedback regarding the CATT for medical professionals. One respondent expressed the following: “Concussion diagnosis is a bit of hit and miss at times. There are still plenty of medical professionals out there who believe concussion is only possible when the person has an episode of unconsciousness. Training as CATT or equivalent should be made mandatory to all healthcare professionals working at emergency but more importantly to physicians and nurses in primary health care.” Another stated: “I do research on concussion. I completed the course in order to evaluate its usefulness for clinicians. I was in the process of reviewing other e-learning courses on concussion that existed in the U.S. I was very impressed with your course and in fact, I believe it is the best such course available anywhere. The developers are to be commended.” Respondents also appreciated that the CATT module is free, not time-consuming, easily accessible, and user-friendly. One respondent explained the following: “I work in the health system in a rural community. Access (physical and financial) to training programs is very limited. The fact that these programs are free and high quality is imperative to being used in rural communities.”

Of the three respondents with negative feedback, one indicated that the information presented in the CATT is redundant to their medical training, however, did indicate that people involved in hockey should learn more about concussion, such as trainers, coaches, and athletes. Another respondent felt that information was missing regarding current research and treatment methods but did not provide specific examples, and the third found the course to be “extremely unsatisfactory” without providing any additional specific information and indicated that the level of concussion knowledge among healthcare professionals is “severely lacking.” The majority of the respondents who reported that they did not learn from the CATT for medical professionals module, nor changed their practice, reported that they already knew the information provided and that the module was useful in confirming that their current practice was appropriate and optimal. For example, one respondent explained that “there was no new information for me. The CATT e-learning confirmed that I was treating/managing patients with concussion appropriate in the “base practice” sense.”

## 4. Discussion

Medical and healthcare professionals have reported a lack of adequate continuing education training and tools for optimal evidenced-based management and evaluation of concussion [25, 26]. Although balance, eye-tracking, and neurocognitive tests exist to assist with the diagnosis of concussion [27, 28], most assessments typically remain solely symptom-based [29]. Optimal management of short- and long-term concussion symptoms can significantly improve patients' quality of life and potentially minimize the financial burden associated with concussion. Up-to-date concussion training for medical and healthcare professionals and access to resources are therefore critical for accurate diagnosis and adequate treatment interventions.

The CATT for medical professionals directly addresses this widely recognized gap in current medical training programs and aims to translate current evidence-based knowledge into best practices in patient care [11, 22, 30]. The primary aim of the current paper was to summarize the general uptake of the CATT amongst medical and healthcare professionals, including results of a QA/QI assessment survey. Overall, results are favourable and provide support that the CATT for medical professionals is a promising evidence-based e-learning resource capable of filling this important gap in concussion training amongst medical and healthcare professionals from different backgrounds and healthcare settings.

The CATT for medical professionals was accessed by a diverse group of professionals within the first two years since its relaunch, and Google Analytics data demonstrated an increase in the use of the CATT in its second year as compared to the first year. This suggests that the CATT for medical professionals is an accessible and acceptable training tool that is increasing in popularity as medical and healthcare professionals become more aware of its availability. Results of the QA/QI assessment indicated favourable results in self-reported change in practice. The majority of respondents reported learning new information about concussion diagnosis, treatment, and management, referring patients to the CATT online resource or distributing CATT materials to patients, and recommending the CATT e-learning to colleagues. Subjective qualitative data of respondents' opinions and experience with the CATT for medical professionals also indicated highly favourable feedback and good overall acceptance. While respondents of the QA/QI assessment all reported residing in Canada, they represented nine provinces and various healthcare systems. This serves as an indicator of the generalizability of the resource as an accepted and appropriate training tool to medical and healthcare professionals across different backgrounds, settings, and regions. This is encouraging with respect to current efforts to expand and promote the use of the CATT for medical professionals worldwide.

The CATT represents a significant effort towards promoting knowledge translation of current concussion care evidence through clear and comprehensive protocol for the diagnosis, treatment, and management of concussion. To our knowledge, CATT for medical professionals is the only accredited online concussion course developed specifically for medical and healthcare professionals, providing practical guidance in the care of concussed patients expanding from addressing youth returning to sports or school, to adults returning to work. Other programs, such as the Centers for Disease Control and Prevention's Heads Up in the US, focus primarily on children and youth in sports.

While improvements in concussion care have been substantial over the past decade, results of a 2018 survey commissioned by the Public Health Agency of Canada indicated that, although 97% of Canadian respondents consider concussion to be an important health issue, half of them had little or no knowledge about concussion and did not know where to go to find information [31]. The importance of adequate knowledge on concussion among the general population is also highlighted by the fact that the identification of concussion relies heavily on subjective reporting [32]. Indeed, concussion education has been strongly associated with both higher reporting rates and decreased risk-taking behavior [33, 34]. In addition to the results of this study, large CATT module completion numbers among medical and healthcare professionals, coaches, parents and caregiver, and workers and their workplace (>60, 000 individuals), distribution numbers (>28, 000 print resources), media appearances (>100 appearances), and mandate numbers (>35 sports associations and schools) are other indicators that the CATT is a feasible means to enhance concussion knowledge for everyone.

Limitations to this study include the self-reported assessment of change in knowledge and in practices, which may have prompted potential response bias. Respondents with favourable opinions about the training may have been more receptive to participating in the study as compared with those who were more neutral or critical. Additionally, respondents actively searching for concussion education may have been more willing to participate than those not interested in further concussion education. This may have introduced a volunteer bias, reducing the generalizability of the results of the current study. Finally, although our survey response rate was similar to these of several previous studies (as documented by Wiebe et al. [35] who acknowledge the declining survey response rates among Canadian clinicians), nonresponse bias inherent in survey design may have resulted in a misrepresentation of the medical and healthcare professional community and influenced the current results. A formal nonresponse bias analysis would have been beneficial. Incentives for participation might have helped improve our response rate [7, 36]. Although respondents of the current study were from many different regions and healthcare systems of Canada, respondents from other countries would have increased the generalizability of the study.

Future directions for the CATT for medical professionals include investigation of opportunities for integration into medical university curricula and continue promotion of the resource in Canada and around the world. Other countries are taking an interest in the educational training of the CATT, such as Kampala, Uganda. In partnership with their local government and sporting federations, training sessions have taken place with medical and healthcare professionals and athletic directors and trainers to increase their knowledge and awareness of concussions. A medical school in Japan is also currently using the CATT resources. Discussions of partnership are also underway with the Faculty of Health Sciences at the University of Beirut in Lebanon to train school nurses, as well as with the Auckland University of Technology in New Zealand to raise awareness and education amongst general practitioners.

## 5. Conclusion

With a growing number of people seeking concussion care, medical and healthcare professionals are increasingly required to diagnose, manage, and when appropriate, refer patients with persistent concussion symptoms to specialized care. Providing up-to-date evidence-based concussion training and resources supports the desired culture changes around concussion awareness and best practices among both medical and healthcare professionals and their patients. The CATT for medical professionals represents an important step forward for knowledge translation and increased use of evidence-based approaches to concussion diagnosis, treatment, and management. In future studies, we will investigate potential predictors and barriers of concussion knowledge uptake and use of best practices by medical and healthcare professionals in order to inform the development of enhanced approaches to knowledge translation.

## Figures and Tables

**Table 1:** Content overview of the CATT for medical professionals, self-assessments throughout.

Core sections and topics	Learning objectives
Introduction: intended audience; purpose; learning objectives; overall course content	

Definition and epidemiology	(A) Understand the anatomy of concussion in order to assess, manage, and refer a patient with a suspected concussion more effectively.
(i) Nature of a concussion	
(ii) Learning check	

Medical assessment	(A) Identify the importance of an early initial assessment. (B) Thoroughly assess a patient with a suspected concussion. (C) Effectively document a concussion with a medical assessment letter.
(i) Medical assessment and clinical history	
(ii) Signs, symptoms, and red flags	
(iii) Physical examination	
(iv) Adjunctive tests	
(v) Documentation	
(Vi) Supplementary resources	
(vii) Learning check	

Management and medical clearance	(A) Manage the initial care of a patient diagnosed with a concussion. (B) Manage a patient's gradual return to activity postconcussion. (C) Understand the considerations involved in return-to-school, return-to-sport, and medical clearance decisions.
(i) Initial rest and symptom management	
(ii) What to inform the patient	
(iii) Return to activity	
(iv) Clinical follow-up and medical clearance	
(v) Documentation	
(Vi) Supplementary resources	
(vii) Learning check	

Persistent symptoms and management	(A) Understand persistent concussion symptoms and associated conditions. (B) Understand treatment and rehabilitation for persistent concussion symptoms and associated conditions. (C) Identify indicators for referral to multidisciplinary care for patients with persistent concussion symptoms.
(i) Persistent symptoms	
(ii) Making referral decisions	
(iii) Other persistent symptoms	
(iv) Supplementary resources	
(v) Learning check	

Physiotherapy and occupational therapy	(A) Support a patient experiencing concussion-related issues through physiotherapy. (B) Support a patient experiencing concussion-related issues through occupational therapy. (C) Assess, develop, and manage a personalized rehabilitation and treatment plan to support a patient during recovery. (D) Recognize indications for referral to other healthcare practitioners.
(i) Role of physiotherapists	
(ii) Role of occupational therapists	
(iii) Supplementary resources	
(iv) Learning check	

Conclusion: postassessment and review; certification; staying up-to-date	

**Table 2:** The CATT for medical professionals website Google Analytics, Year 1 June 11, 2018, to May 31, 2019, and Year 2 June 1, 2019, to May 31, 2020.

CATT component		Pageviews	
		Year 1	Year 2
Medical professional landing page	Pageviews	8,072	9,382
SCAT5 landing page	Pageviews	6,083	7,009
SCAT5 tool	Visits	18,611	24,704
Child SCAT5 tool	Visits	1,523	3,058
Course completions (English and French)		575	628

**Table 3:** QA/QI respondents' demographics (*n* = 89).

Demographics	Proportion % (*n*)
Time since taking CATT	
1–3 months	16.9 (15)
4–6 months	35.9 (32)
7–12 months	33.6 (30)
>12 months	13.5 (12)

Source of hearing about CATT	
Referred from a colleague	32.6 (29)
Internet search	22.5 (20)
e-mail	19.1 (17)
Browsing cattonline.com	7.9 (7)
Social media	5.6 (5)
Newsletter	4.5 (4)
Others	22.5 (20)

**Table 4:** QA/QI self-reported learning and behavior following completion of the redeveloped CATT for medical professionals (*n* = 89).

Have you...	Proportion % (*n*)
Learned new information?	
Yes	55.1 (49)
Somewhat	30.3 (27)
No	14.6 (13)

Changed your practice? (if applicable, *n* = 66)	
Yes	30.3 (20)
Somewhat	42.4 (28)
No	27.3 (18)

Accessed SCAT5/Child SCAT5 from the CATT? (if applicable, *n* = 67)	
Yes, often	31.3 (8)
Yes, sometimes	23.9 (22)
No, I access them elsewhere	32.8 (16)
No, I do not use them	11.9 (21)

Recommended the CATT to colleagues?	
Yes	70.5 (62)
No	29.5 (26)

Referred patients to the CATT? (if applicable, *n* = 65)	
Yes	47.7 (31)
Sometimes	23.1 (15)
No	29.2 (19)

## Data Availability

Requests for access to the data used to support the findings of this study should be made to Dr. Shelina Babul, Associate Director, Sports Injury Specialist, BC Injury Research and Prevention Unit, BC Children's Hospital-sbabul@bcchr.ca.

## Appendix

### A. Program Planning Committee

  Dr. Shelina Babul, PhD, Associate Director, Sports Injury Specialist, BC Injury Research and Prevention Unit, BC Children's Hospital; Investigator, BC Children's Hospital Research Institute; Investigator, Djavad Mowafaghian Centre for Brain Health, UBC; Clinical Associate Professor, Dept. of Pediatrics, Faculty of Medicine, UBC
  Dr. Shannon Bauman, MD, CCFP (SEM), Dip. Sport Med, Medical Director, Concussion North; Dept. of Family Medicine, Dept. of Surgery, Royal Victoria Regional Health Centre
  Dr. Michael Ellis, MD, FRCSC, Medical Director, Pan Am Concussion Program; Dept. of Surgery and Pediatrics and Section of Neurosurgery, University of Manitoba; Scientist, Children's Hospital Research Institute of Manitoba; Co-Director, Canada North Concussion Network
  Ms. Pamela Fuselli, MSc, VP, Knowledge Transfer and Stakeholder Relations, Parachute
  Dr. Paul Korn, MD, FRCPC, Medical Director, Emergency Department, BC Children's Hospital; Clinical Associate Professor, Department of Pediatrics, UBC
  Dr. Nick Reed, PhD, MScOT, OT Reg (Ont), Clinician Scientist, Bloorview Research Institute; Co-Director, Concussion Centre, Holland Bloorview Kids Rehabilitation Hospital; Assistant Professor, Dept. of Occupational Science and Occupational Therapy, University of Toronto
  Ms. Jennifer Scarr, RN, MSN, Provincial Lead, Health Promotion, Prevention and Primary Care, Child Health BC
  Dr. Charles Tator, MD, FRCSC, Professor of Neurosurgery, University of Toronto; Division of Neurosurgery and Canadian Concussion Centre, Toronto Western Hospital

### B. CATT References

  Davis et al. (2017). What is the difference in concussion management in children as compared with adults? A systematic review. Br J Sports Med, 51 (12), 949–957.
  Feddermann-Demont et al. (2017). What domains of clinical function should be assessed after sport-related concussion? A systematic review. Br J Sports Med, 51, 903–918.
  Makdissi et al. (2017). Approach to investigation and treatment of persistent symptoms following sport-related concussion: a systematic review. Br J Sports Med, 51 (12), 958–968.
  McCrory et al. (2017). Consensus statement on concussion in sport: the 5^th^ international conference on concussion in sport held in Berlin, October 2016. Br J Sports Med, 51 (11), 838–847, http://www.bjsm.bmj.com/content/51/11/838.
  Ontario Neurotrauma Foundation (2017). Standards for postconcussion care, http://www.concussionsontario.org.
  Ontario Neurotrauma Foundation (2018). Guideline for Concussion/Mild Traumatic Brain Injury and Persistent Symptoms, 3^rd^ edition. Toronto: ONF, https://braininjuryguidelines.org/concussion/.
  Parachute (2017). Canadian Guideline on Concussion in Sport. Toronto: Parachute, http://www.parachutecanada.org/guideline.
  Reed, N., Zemek, R., Dawson, J., Ledoux, AA. et al. (2019). Living Guideline for Diagnosing and Managing Pediatric Concussion. Toronto: Ontario Neurotrauma Foundation, https://braininjuryguidelines.org/pediatricconcussion/.
  Schneider et al. (2017). Rest and treatment/rehabilitation following sport-related concussion: a systematic review. Br J Sports Med, 51 (12), 930–934.

### C. Organizations That Promoted the Relaunch of the CATT for Medical Professionals

  Parachute
  BC Injury Research and Prevention Unit
  BC Children's Hospital Research Institute
  Alberta Injury Prevention Centre
  BC Concussion Advisory Network
  Doctors of BC
  Child Health BC
  UBC Faculty of Medicine
  Djavad Mowafaghian Centre for Brain Health (UBC)
  BC College of Family Physicians
  BC Medical Journal
  BC Ministry of Health
  BC Provincial Health Services Authority
  Vancouver Coastal Health
  VGH Trauma Services
  Physiotherapy Association of BC
